# Unusual glomus tumor of the bladder: a rare case report and literature review

**DOI:** 10.1186/s12894-021-00837-0

**Published:** 2021-04-21

**Authors:** Li Chen, Bin Lai, Xiaoyan Su, Jiwei Wang

**Affiliations:** 1 Department of Ultrasound, The Second Affiliated Hospital of Nanchang University, Nanchang, 330006 Jiangxi China; 2 Department of Gastrointestinal Surgery, The Second Affiliated Hospital of Nanchang University, No1 Mingde Road, Nanchang, 330006 Jiangxi China; 3 Department of Pathology, The Second Affiliated Hospital of Nanchang University, Nanchang, 330006 Jiangxi China

**Keywords:** Glomus tumor, Bladder, Urinary tract

## Abstract

**Background:**

Glomus tumor (GT), which are neoplasms of the glomus body, usually occur in the extremities, particularly under the nail bed. GT occurring in the bladder is very rare and has been reported as sporadic. In the present study, a rare case of bladder GT is reported and its clinical and histopathological characteristics are summarized by literature review.

**Case presentation:**

A 57-year-old woman presented with intermittent gross hematuria for 2 years. Urinalysis displayed hematuria. The bladder ultrasound showed an avascular and homogeneous isoechoic polypoid mass with a maximum diameter of 6 mm at the right lateral wall of bladder. The bladder endoscopic examination showed a polypoid lesion, with a smooth surface, located in the right lateral wall. Then, a transurethral resection was performed, its histopathological features indicated a benign GT.

**Conclusions:**

GT arising in the bladder is extremely rare, and only four cases have been identified in studies reported in English. It is difficult to diagnose bladder GTs according to their clinical features. The gold standard method used for their diagnosis is histopathology. However, it should also be considered in the differential diagnosis for bladder mass.

## Introduction

Glomus tumor (GT) is a mesenchymal neoplasm, composed of a mixture of glomus cells, blood vessels and smooth muscle cells arising from the glomus body. GT can occur in almost any part of the body, but it is most commonly seen in the extremities, particularly in the nail bed [1]. GT occurring in the bladder is very rare and has been reported sporadically. To the best of our knowledge, only 4 cases of bladder GT have been previously identified in studies reported in English [2–5]. The present study aimed to (1) present an extremely rare case of GT in the bladder and (2) summarize its clinical and histopathologic features by literature review.

## Case presentation

**Fig. 1:**
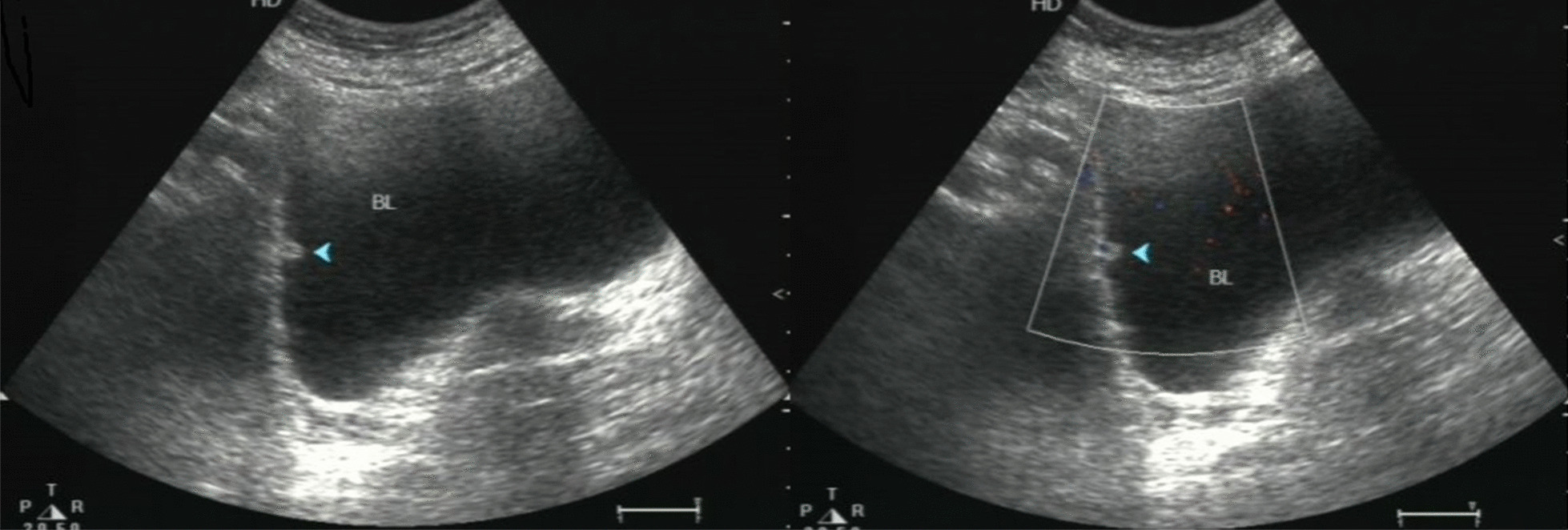
Bladder ultrasound indicated a homogeneous isoechoic polypoid mass with a maximum diameter of 6 mm at the right lateral wall of bladder

**Fig. 2:**
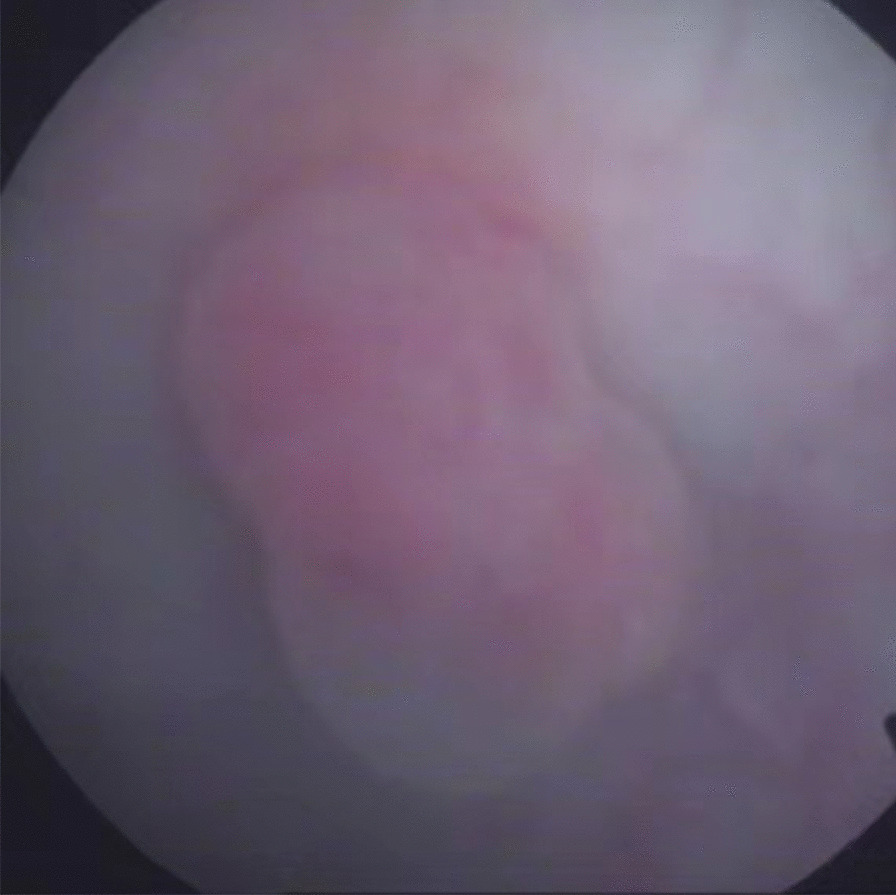
Cystoscopy examination revealed a polypoid lesion with a smooth surface, located in the right lateral wall of bladder

**Fig. 3:**
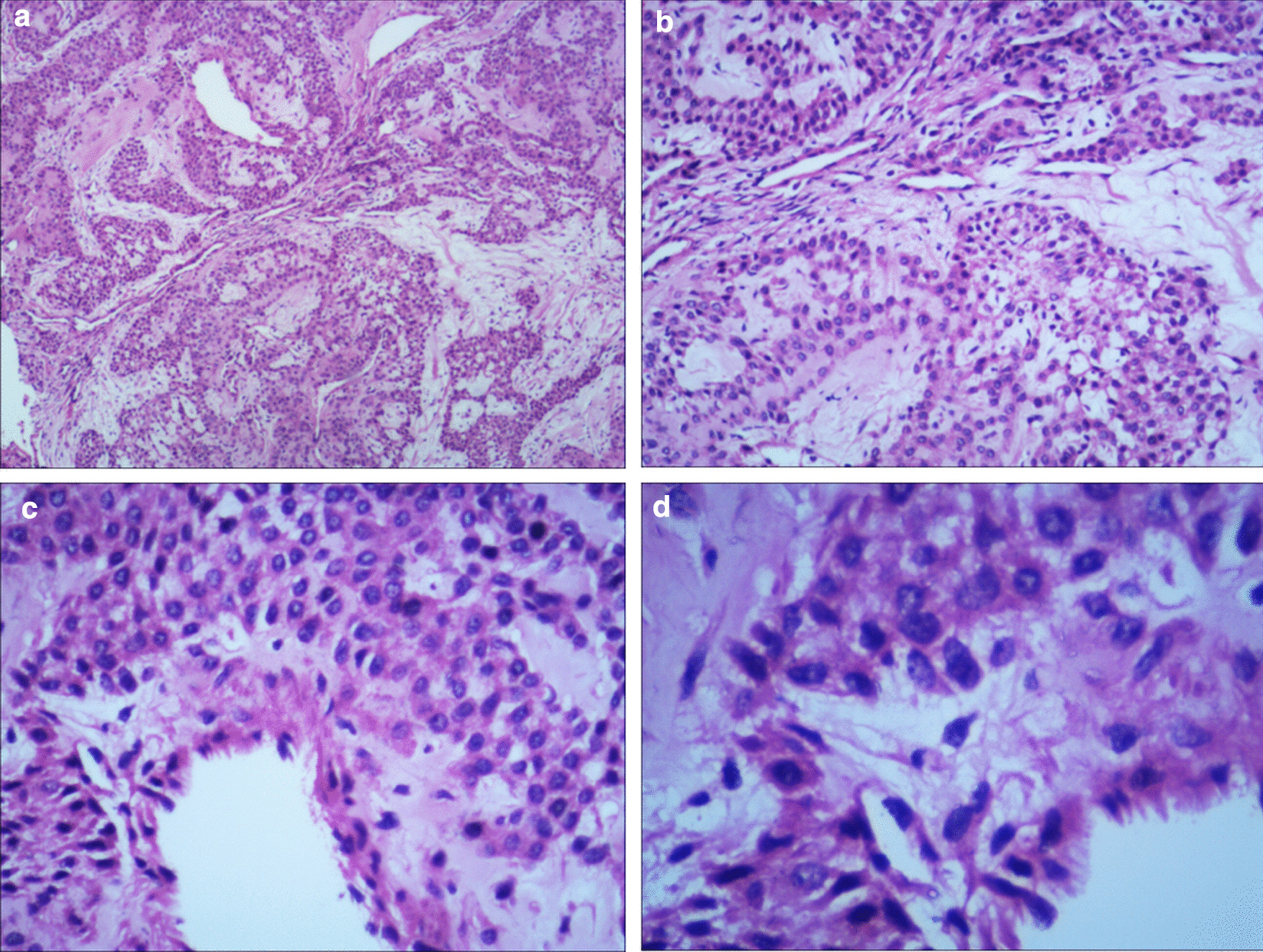
Microscopically, the neoplasm was well circumscribed and composed of nests of monomorphic cells with bland nuclei and eosinophilic cytoplasm, clustered around dilated vessels (hematoxylin-eosin, original magnifications ×25 (**a**), ×100 (**b**), ×200 (**c**), and ×400 (**d**))

**Fig. 4:**
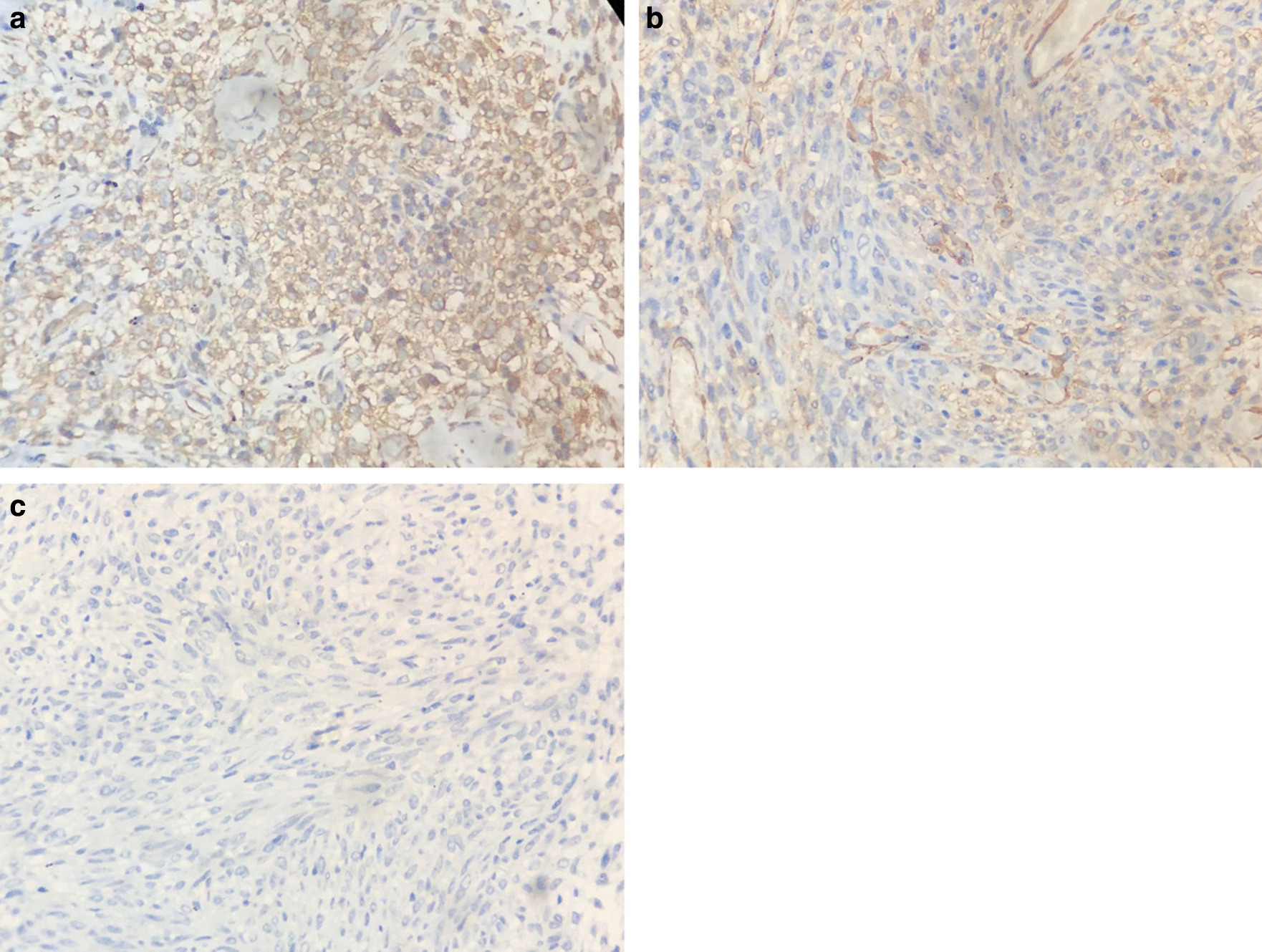
Immunohistochemistry demonstrated that neoplastic cells exhibited positive reactivity to vimentin (**a**) and smooth muscle actin (**b**) and negative to desmin (**c**). (original magnifications ×200)

A 57-year-old woman presented with intermittent gross hematuria for 2 years. She did not report any other symptoms. Physical examination indicated no abnormal findings. Blood cell counts and biochemical tests were within the reference range. Urinalysis displayed hematuria. Therefore, it was suggested that she should undergo a urinary system ultrasound examination. The kidney and ureter ultrasounds indicated no abnormal findings, whereas the bladder ultrasound revealed an avascular and homogeneous isoechoic polypoid mass with a maximum diameter of 6 mm at the right lateral wall of the bladder (Fig. 1). She has no bladder tumors history or family history. So, the bladder of the patient was examined endoscopically and a polypoid lesion (6 mm in maximum diameter) was noted with a smooth surface, located in the right lateral wall (Fig. 2). Subsequently, a transurethral resection (TUR) was performed and the lesion was easily removed. Microscopic examination indicated that the neoplasm was well circumscribed and composed of nests of monomorphic cells with bland nuclei and eosinophilic cytoplasm, clustered around dilated vessels. Atypia, mitoses, intravascular growth and necrosis were absent (Fig. 3). Immunohistochemical staining revealed that the neoplastic cells reacted positively to the smooth muscle actin (SMA) and vimentin, whereas they were negative to desmin (Fig. 4). A diagnosis of benign bladder GT was made. The patient remained asymptomatic and no recurrence was observed within a 2-year follow up.

## Discussion and conclusions

GT, which are neoplasms of the glomus body, usually occur in the extremities, particularly under the nail bed. Localizations other than the extremities have been reported in various systems including the respiratory (e.g. nose, trachea, lung) [6–8] the digestive (e.g. larynx, esophagus, stomach, intestine, liver) [9–13], the reproductive (e.g. uterine cervix, ovary, testis) [14–16], the urinary (e.g. kidney, bladder, urethra) [2–5, 17, 18], the endocrine (e.g. thyroid, breast) [19, 20], the nervous (e.g. sciatic nerve) [21] and the cardiovascular (e.g. heart, carotid artery) [22, 23]. However, these are uncommon.

GT occurring in the bladder is very rare and has been reported as sporadic. To the best of our knowledge, only 4 cases of human bladder GT have been identified in the literature, indicating that the current case is the fifth. The clinical data of these five patients are presented in Table 1 and the histopathological data in Table 2. After reviewing of the clinical features of these patients, the following conclusions were made: (1) Bladder GT can occur in males (M) and females (F), no significant gender difference was evident; (2) Bladder GT mostly occurred in elderly subjects, with an age range from 56 to 84 years; (3) Bladder GT patients presented with hematuria or were asymptomatic; (4) Bladder GT patients usually exhibited no history of bladder tumor; (5) The size and location of bladder GT were flexible (the size ranged from 3 to 65 mm and the location could be anterior, lateral, or posterolateral wall); (6) Although the majority of GTs are benign, the bladder GTs were described in the present and previous studies were not always benign (2/5 cases were benign, 2/5 cases were atypical and 1/5 case was malignant); (7) Transurethral resection is the most common treatment used for benign or atypical bladder GT, whereas for malignant bladder GT, comprehensive treatment could be performed according to patient condition. Although these findings are meaningful, no specific clinical feature was noted. The pathological and immunohistochemical examination are the standard methods used in the diagnosis of bladder GT.

**Table 1:** Clinical data from previous cases and the present case of glomus tumor in the bladder

Case no	Authors and reference	Age (yr.)	Sex	Cause of clinic visiting	Bladder tumor history	First-detected technique	Tumor size (mm)	Tumor location	Biochemistry and hematologic test	Diagnosis	Treatment	Follow-up/clinical result
1	Shim HS, et al^4^	57	F	Gross hematuria	No	Magnetic resonance imaging	65	Left lateral wall	N/A	Malignant GT	TUR + chemotherapy	2 months/Died
2	Tripodi SA, et al^3^	63	M	Gross hematuria	No	Cystoscopy	12	Anterior wall	Within the reference range	Benign GT	TUR	1 year/Free
3	Lindsay LW, et al^5^	84	F	Incidental finding	low grade superficial urothelial carcinoma	Cystoscopy	3	Right posterolateral wall	N/A	Atypical GT	TUR	N/A
4	Palmisano F, et al^2^	58	M	Incidental finding	No	Computed tomography	25	Anterior wall	Within the reference range	Atypical GT	Robot- assisted Partial Cystectomy	7 months/Free
5	Jiwei W, et al. (present case)	56	F	Gross hematuria	No	Ultrasound	6	Right lateral wall	Within the reference range	Benign GT	TUR	1 year/Free

F, female; M, male; N/A, not mentioned in literature; GT, glomus tumor; TUR, transurethral resection

**Table 2:** Pathological data from previous cases and the present case of glomus tumor in the bladder

Case No	Authors and reference	Pathologic features	Immunohistochemically stains	
			Positive	Negative
1	Shim et al^4^	A large number of tumor cells had infiltrated into the subepithelial connective tissues and proper muscles, which were primarily perivascular in distribution. The cells showed diffuse cytologic atypia with spindle morphology and marked nuclear atypia with high mitotic activity (50/10HPF); Multifocal tumor necrosis and hemorrhage were also noted	SMA	Cytokeratins, Epithelial membrane antigen, S100 protein, Desmin, CD31, CD34
2	Tripodi et al^3^	The neoplasm was well circumscribed and composed of nests of monomorphic cells with bland nuclei and eosinophilic cytoplasm. Atypia, mitoses, intravascular growth, and necrosis were absent	SMA, CD34	p63, Cytokeratin AE1/AE3
3	Lindsay et al^5^	Abnormal proliferation of ovoid cells in the lamina propria, uniform ovoid nuclei with indistinct eosinophilic cytoplasm, sheet-like growth pattern with a prominent capillary network, mild nuclear atypia and mototic index accounted to 2 mitosis/HPF	SMA, Smooth muscle myosin	CD 34
4	Palmisano et al^2^	The neoplasm was composed of uniform small oval to spindle cells, without nuclear atypias, disposed in a vaguely storiform pattern, separated by a vascular stroma; numerous cells with bizarre nuclei were scattered throughout the lesion; mototic index accounted to 2 mitosis/50HPF	SMA, Vimentin, bcl-2	Citokeratins, p63, Desmin, Calponin, CD34, S100, HMB-45, CD68R, c-kit, DOG-1, ALK-1, Chromogranin, a-Inhibin
5	Jiwei et al. (present case)	The tumor cells were uniform small oval and clustered around dilated vessels. Atypia, mitoses, intravascular growth, and necrosis were absent	SMA, Vimentin	Desmin

HPF, high power fields; SMA, Smooth muscle actin

GT is composed of a mixture of glomus cells, blood vessels and smooth muscle cells. GT is usually benign and rarely malignant or atypical. The criteria of malignancy GT are the following: (1) tumor with a deep location, (2) a size more than 2 cm, (3) atypical mitotic figures or apparent nuclear atypia, (4) 5 or more mitotic figures/50 high-power field [24]. The atypical GT was defined as a tumor with a high-grade nuclear pleomorphism in the absence of any other malignant features, such as large size, deep location, infiltrative growth, mitotic activity, or necrosis. The majority of the GTs are benign, whereas this not commonly noted for bladder GT. According to the present literature review, more than half of the patients with bladder GT were malignant or atypical cases.

In conclusion, GT arising in the bladder is extremely rare, and only four cases have been identified in studies reported in English. It is difficult to diagnose bladder GTs according to their clinical features. The gold standard method used for their diagnosis is histopathology. However, it should also be considered in the differential diagnosis for bladder mass.

## Data Availability

Records and data pertaining to this case are in the patient’s secure medical records in the Second Affiliated Hospital of Nanchang University. All searched data by literature review are included in this paper.

## Abbreviations

GT: Glomus tumor
TUR: Transurethral resection
HPF: High power fields
SMA: Smooth muscle actin
F: Female
M: Male
N/A: Not mentioned or available

## References

[1] Vieira, F. G., Nakamura, R., Costa, F. M., Canella, C., & Marchiori, E. (2016). Subungual glomus tumor. J Clin Rheumatol 22(6), 331. 10.1097/RHU.0000000000000418 27556243

[2] Palmisano F, Gadda F, Spinelli MG (2018). Symplastic glomus tumor of the urinary bladder treated by robot-assisted partial cystectomy: a case report and literature review. Urologia.

[3] Tripodi SA, Rocca BJ, Mourmouras V (2013). Benign glomus tumor of the urinary bladder. Arch Pathol Lab Med.

[4] Shim HS, Choi YD, Cho NH (2005). Malignant glomus tumor of the urinary bladder. Arch Pathol Lab Med.

[5] Waters LL, Zhai QH, Buie JS (2010). Atypical glomus tumor of uncertain malignant potential in the urinary bladder. Pathol Lab Med Int.

[6] Meguro S, Kusama Y, Matsushima S (2019). Nasal glomus tumor: A rare nasal tumor with diffuse and strongly positive synaptophysin expression. Pathol Int.

[7] Wang C, Ma Y, Zhao X (2017). Glomus tumors of the trachea: 2 case reports and a review of the literature. J Thorac Dis.

[8] Wan PZ, Han Q, Wang EH (2015). Glomus tumor of uncertain malignant potential of the lung: a case report and review of literature. Int J Clin Exp Pathol.

[9] Aslam N, Qazi ZU, Ahmad AH (2012). Malignant glomus tumour of larynx: first case report and literature review. J Laryngol Otol.

[10] Segura S, Mansoor S, Gorelick AB (2015). Glomus tumor of the esophagus: a case report and review of the literature. Conn Med.

[11] Masouminia M, Ghani HA, Foote D (2018). Rare presentation of the glomus tumor in the stomach. Exp Mol Pathol.

[12] Campana JP, Goransky J, Mullen EG (2014). Intestinal benign glomus tumor: description and review of the literature. Dig Dis Sci.

[13] Aversa JG, Monroe C, Levi A (2020). The first malignant primary hepatic glomus tumor: a case report. Int J Surg Case Rep.

[14] Aynardi JT, Kim SH, Barroeta JE (2016). Epithelioid glomus tumor of the uterine cervix: a case report and review. Int J Gynecol Pathol.

[15] Maeda D, Takazawa Y, Oda K (2010). Glomus tumor of the ovary: a case report. Int J Surg Pathol.

[16] Tullie STE, Quraishi MK, Karawita T (2019). Rare presentation of a testicular glomus tumour. BMJ Case Rep.

[17] Chen YA, Li HN, Wang RC (2017). Malignant glomus tumor of the kidney: a case report and review of the literature. Clin Genitourin Cancer.

[18] Ramsay S, Chan G, Zimmerman WB, Chee J (2019). Glomus tumour of the male urethra: an unusual diagnostic. BMJ.

[19] Liu Y, Wu R, Yu T (2019). Malignant glomus tumor of the thyroid gland: a case report. J Int Med Res.

[20] Mizutani L, Tanaka Y, Kondo Y (2014). Glomus tumor of a female breast: a case report and review of the literature. J Med Ultrason.

[21] Nwankwo BO, Henshaw RM, Kumar D (2018). Glomus tumor of the sciatic nerve: an extraspinal cause of sciatica. Orthopedics.

[22] Elkrinawi R, Usta E, Baumbach H (2012). Late recurrence of a cardiac glomus tumor. Thorac Cardiovasc Surg.

[23] Satis S, Tuna M, Alparslan N (2019). Jarcho-Levin syndrome and concomitant carotid glomus tumor: first reported case. World Neurosurg.

[24] Gill J, Van Vliet C (2010). Infiltrating glomus tumor of uncertain malignant potential arising in the kidney. Hum Pathol.

